# Mr. Nathaniel Rochester

**Published:** 1906-03

**Authors:** 


Mr. Nathaniel Rochester, of Buffalo, died at his home March 5, 1906, aged 52 years. He was the elder son of the late Dr. Thomas F. Rochester, and married some years ago, Miss Hauenstein, daughter of the late Dr. John Hauenstein. Mr. Rochester, at the time of his death, was president of the Third National Bank of Buffalo, an office he had held for several years. He was concededly one of the first citizens of Buffalo and his presence will be missed in many relations which he bore to the community,—in church, social, business and general affairs he was a man of influence whom the city can ill afford to lose.

